# Psychological morbidity in Nepali cross-border migrants in India: a community based cross-sectional study

**DOI:** 10.1186/s12889-019-7881-z

**Published:** 2019-11-15

**Authors:** Raja Ram Dhungana, Nirmal Aryal, Pratik Adhikary, Radheshyam Krishna KC, Pramod Raj Regmi, Bikash Devkota, Guna Nidhi Sharma, Kolitha Wickramage, Edwin van Teijlingen, Padam Simkhada

**Affiliations:** 1 Green Tara Nepal, Kathmandu, Nepal; 2 Institute for Health & Sport (IHES), Victoria University, Melbourne, Australia; 3 Faculty of Health & Social Sciences, Bournemouth University, Bournemouth, UK; 4 School of Public Health, University of California, Berkeley, USA; 5 International Organization for Migration, Kathmandu, Nepal; 6 Ministry of Health and Population, Kathmandu, Nepal; 7 International Organization for Migration, Geneva, Switzerland; 8 International School of Health and Human Science, The University of Huddersfield, Huddersfield, UK

**Keywords:** Migrant workers, Prevalence, Psychological morbidity, India, Nepal

## Abstract

**Background:**

Since Nepali cross-border migrants can freely enter, work and stay in India, they are largely undocumented. The majority is involved in semi-skilled or unskilled jobs with limited labour rights and social security, a fact which predisposes them to psychological distress. We aimed to assess the prevalence of and factors associated with psychological morbidity among Nepali migrants upon their return from India.

**Methods:**

A community based cross-sectional study was conducted in six districts of Nepal between September 2017 and February 2018. A total of 751 participants who had worked at least six months in India and returned to Nepal were interviewed from 24 randomly selected clusters. The General Health Questionnaire (GHQ)-12 was used to measure the psychological morbidity. Data were analysed using Poisson regression analysis.

**Results:**

The majority was younger than 35 years (64.1%), male (96.7%), married (81.8%), had at least a primary education (66.6%), and belonged to Dalit, Janajati and religious minorities (53.7%). The prevalence of psychological morbidity was 13.5% (CI: 11.2–16.1%). Participants aged 45 years and above (adjusted prevalence ratio (aPR) = 2.74), from the Terai (aPR = 3.29), a religious minority (aPR = 3.64), who received no sick leave (aPR = 2.4), with existing health problems (aPR = 2.0) and having difficulty in accessing health care (aPR = 1.88) were more likely than others to exhibit a psychological morbidity.

**Conclusion:**

This study demonstrated that psychological morbidity was prevalent in the study participants and varied significantly with individual characteristics, work conditions and health. Multifaceted approaches including psychological counselling for returnees and protection of labour and health rights in the workplace are recommended to help reduce psychological morbidity.

## Background

Migrant workers are likely to experience adverse conditions that can influence their health and wellbeing in every phase of migration [1]. Post migration experiences such as exploitation, lack of legal protection, broken social networks, poor health care and discrimination in the destination country can lead to mental illness [1]. Post migration mental health vulnerability increases if migration happens through informal channels or without proper documentation [2–5]. Nepali cross-border migrants to India could also be at risk of psychological morbidity due to irregular migration, lack of legal and social protections [6], and precarious working and living conditions [7, 8]. However, there is scant information on the health and well-being of Nepali migrant workers in general [9] and on the cross-border migrants to India in particular. This study aimed to assess the magnitude and distribution of psychological morbidity in Nepali migrants returning from India.

### Context: open border, undocumented migration to India

Nepal and India share an open border across which citizens can move freely without legal restrictions. This fact, combined with strong sociocultural affinity, has made India a major destination for Nepali labour migrants since the Anglo-Nepal war of 1814–15 [10]. India’s recent economic growth and the fact that it offers better wages and employment opportunities than Nepal have acted as major pull factors, particularly for Nepali workers living in mid- and far-western border districts [7, 11]. Poverty, unemployment and low wages are the most cited push factors for cross-border migration to India [7, 11].

Since neither registration nor obtaining labour permission is required for those going to India, cross-border migration is largely undocumented and, hence poorly understood [10]. Different studies and reports provide different estimates of the number of Nepali living in India, ranging from 0.5 to 3 million [6]. Recent estimates suggest that between 17% [12] and 37.6% [13] of all Nepali migrants choose India as their destination. The majority of Nepali migrants to India were males (84%) [13] and had either no or only primary education (68.3%) [7]. Most were either security guards (48.6%), restaurant workers (13.1%) or wage labourers (12.3%) [7]. Generally, it is the poorest, most marginalised semi-skilled or unskilled Nepali workers who migrate from neighbouring districts in Nepal to India for work [8, 11].

Long delay in paying wages, unpaid overtime, lack of health insurance schemes, long working hours, lack of safety and security measures and poor hygiene are the major work, living and health related problems frequently encountered by Nepali workers in India [7]. Since most Nepali migrants are involved in the informal economy primarily as domestic workers, security guards, porters, coal miners, tea plantation and other farm workers and have no access to legal rights or security, it is claimed that Nepali migrants are taken as granted and are vulnerable to many forms of exploitation [6].

### Migration and mental health

The preponderance of evidence suggests the migration is a stress-inducing phenomenon [14–16]. The migration process is associated with many complex inter-relating psychological, social and cultural factors of both an individual and a collective nature [4, 17], all of which negatively affect people’s psychological wellbeing [15, 18]. Studies have demonstrated that non-migrant specific factors such as being female, poor socio-economic status, low educational attainment and existing health conditions predict poor mental health in migrants [2, 15]. Similarly, migrants who experienced abuse or exploitative treatment or perceived a lack of safety and security in the workplace had an increased risk of mental illness [14, 17, 19]. Additionally, cultural incongruity, alienation, lack of social support and migration related family stress during and after migration appear to make migrant workers more vulnerable to psychological distress [2, 4, 17].

Studies have found that migrants sometimes adopt coping strategies and develop resilience to address mental health stressors. Migrants who established their cultural identity and had socio-cultural support mechanisms were more able to avoid mental health threats in host countries [14, 19], a fact which signifies the importance of neighbourhood ethnic density, social cohesion and support and a smooth process of acculturation for protecting the psychological wellbeing in migrants [15, 20].

## Methods

### Data source, study participants and sampling

This study is part of a larger survey titled ‘Health Vulnerabilities of Cross-border Migrants’ [21]. The cross-sectional survey was carried out in 2017–2018 with the support of the International Organization for Migration (IOM), Nepal and Green Tara Nepal (GTN) to explore the health vulnerabilities including psychological burden of cross-border migrants from Nepal.

The survey sample was representative of cross-border returnee migrants to India from Nepal. The study participants consisted of 751 returnee migrants who had worked at least six months in India before returning to Nepal. Initially, 24 clusters were selected randomly from Achham, Doti, Kailali, Kanchanpur, Banke, and Surkhet districts. In the absence of a sampling frame, the survey applied multiple non-probability sampling methods to maximize the selection of all households with returnee migrants in each cluster. When there were multiple eligible participants within a household, the survey applied the Kish grid method [22] to select just one individual. Details of the sampling procedure are mentioned elsewhere [21]. The survey obtained ethical clearance from the Ethical Review Board of Nepal Health Research Council and the participants provided written consent before data collection. The response rate of the participants was 99.3%.

### Data collection

The survey used a self-administered questionnaire to collect information about socio-demographic characteristics, work and living conditions, and health and behaviour from returnee migrants [21]. To assess psychological morbidity, the survey applied the General Health Questionnaire-12 (GHQ-12), which has been validated in Nepali [23]. Psychological morbidity was defined as the symptomatic presence of non-specific mental health problems, including psychological distress.

Initially, we dichotomized the GHQ-12 item score as ‘0’ for 0 and 1, and ‘1’ for 2 and 3 and aggregated each item value to get the overall score [23]. We defined the ‘case-ness’ of psychological morbidity based on a GHQ score six and above [23] and re-grouped age, marital status, ethnicity and other variables [Table 1]. We also extracted the standard definition for categorizing current smoking (smoking on at least one day during the past 30 days) [24] and current alcohol use (those who had had at least one drink of alcohol during the 30 days before the survey) [25] from the survey.

**Table 1:** Association of socio-demographic and related factors with psychological morbidity

Characteristics		Frequency (%)	Psychological morbidity		*p* value
		Total^¶^	Non-case-ness	Case-ness	
		n (%)	n (%)	n (%)	
Age	< 25 years	170 (22.7)	148 (87.06)	22 (12.94)	< 0.001
	25–34 years	310 (41.39)	283 (91.29)	27 (8.71)	
	35–44 years	190 (25.37)	164 (86.32)	26 (13.68)	
	45 years and above	79 (10.55)	53 (67.09)	26 (32.91)	
Gender	Male	726 (96.67)	633 (87.19)	93 (12.81)	< 0.001
	Female	25 (3.33)	17 (68.0)	8 (32.0)	
Marital status	Unmarried	123 (16.42)	104 (84.55)	19 (15.45)	0.006
	Married	613 (81.84)	538 (87.77)	75 (12.23)	
	Others (widow, divorced)	13 (1.74)	6 (46.15)	7 (53.85)	
Education	No Schooling	102 (13.58)	75 (73.53)	27 (26.47)	< 0.001
	Primary and lower (1–5 grades)	236 (31.42)	200 (84.75)	36 (15.25)	
	Lower Secondary (6–8 grades)	231 (30.76)	211 (91.34)	20 (8.66)	
	Secondary (9–10 grades)	138 (18.38)	123 (89.13)	15 (10.87)	
	SLC and Higher	44 (5.86)	41 (93.18)	3 (6.82)	
Ethnicity	Brahmin/Chhetri/Thakuri	345 (46.25)	315 (91.3)	30 (8.7)	< 0.001
	Dalit and Janajatis Hill	238 (31.9)	222 (93.28)	16 (6.72)	
	Dalit, Janajatis and others Terai	144 (19.3)	99 (68.75)	45 (31.25)	
	Religious minorities	19 (2.55)	12 (63.16)	7 (36.84)	
Occupation	Hotel worker	209 (28.32)	191 (91.39)	18 (8.61)	< 0.001
	Factory worker	208 (28.18)	172 (82.69)	36 (17.31)	
	Watchmen	197 (26.69)	165 (83.76)	32 (16.24)	
	Others^¶¶^	124 (16.8)	110 (88.71)	14 (11.29)	
Household income (monthly) (in ^§^Nepali Rupee)	< 10,000 NR	128 (17.2)	104 (81.25)	24 (18.75)	0.035
	10,000–19,999 NR	252 (33.87)	221 (87.7)	31 (12.3)	
	20,000–29,999 NR	235 (31.59)	215 (91.49)	20 (8.51)	
	30,000 NR and more	129 (17.34)	105 (81.4)	24 (18.6)	
Salary (monthly) (in Indian Rupee^§§^)	< 10,000 IR	202 (27.08)	166 (82.18)	36 (17.82)	0.03
	10,000–15,000 IR	469 (62.87)	411 (87.63)	58 (12.37)	
	15,001 IR and more	75 (10.05)	70 (93.33)	5 (6.67)	
Was with family^¶¶¶^	Yes	119 (16.35)	98 (82.35)	21 (17.65)	0.15
	No	609 (83.65)	531 (87.19)	78 (12.81)	

Note: ^¶^, Given the column wise total proportion to compare the frequency distribution within each group; ^¶¶^, other occupations were house servant, driver, agriculture workers; ^¶¶¶^, the question asked was ‘Did you use to live with your family member while staying in India?”; ^§,^ 1 Nepali Rupee = 0.0089 USD; ^§§^, 1 Indian Rupee = 0.014 USD

### Data analysis

We performed data analysis using STATA Version 15.1 (Stata Corporation, College Station, TX, USA). Cases with missing values were deleted listwise. All estimates are presented with 95% confidence intervals (CIs). We tabulated findings related to the distribution of psychological morbidity across socio-demographic, work and living conditions, and health and behavioural factors and used the Chi-square test to measure their association. To better understand the differences in the prevalence of psychological morbidity, we used Poisson regression analysis and reported the unadjusted/adjusted prevalence ratio (uPR/aPR) [26]. A *p*-value < 0.05 is considered statistically significant. The independent variables that were significant (p-value < 0.05) in univariate analysis were included in a multivariable model. As the household income had a strong correlation with the salary of the individual, the former variable was excluded from the adjusted model. After the listwise exclusion of the missing data, 571 out of 751 observations were available for multivariable analysis.

## Results

### Demographic of sample

The mean (standard deviation) and median (interquartile range) age of the participants were 32 years (9.2 years) and 31 years (25–38 years) respectively. Participants were overwhelmingly male (96.7%) and a very high proportion (81.8%) were married [Table 1]. Two thirds (66.6%) had completed at least primary school as their highest level of education, and 13.6% had no formal schooling. Just over half (53.7%) belonged to Dalit, Janajati, religious minority or disadvantaged caste groups. Most (83.2%) fitted into one of three job-types: hotel worker, factory worker and guard [Table 1]. The majority of participants had household incomes (51.1%) and salaries (72.5%) less than 20,000 Nepali rupees (NR) or USD 178 per month (exchange rate, 1 NR = 0.0089 USD).

### Work and health conditions

Three quarters of the participant (75.5%) had worked more than eight hours a day and a large minority had had no day off (46%) or sick leave provision (35.9%) [Table 2]. Most participants (97.6%) were either on vacation or had returned for personal reasons. The majority of the returnee migrants had spent less than two years in India during their last stay and were interested in going back again [Table 2].

**Table 2:** Association of work and health related factors with psychological morbidity

Characteristics		Frequency (%)	Psychological morbidity		p value
		Total^¶^	Non-case-ness	Case-ness	
		n (%)	n (%)	n (%)	
Working hours/day	8 h and less	184 (24.5)	161 (87.5)	23 (12.5)	0.66
	> 8 h	567 (75.5)	489 (86.24)	78 (13.76)	
Day off	Yes	341 (45.96)	299 (87.68)	42 (12.32)	0.39
	No	401 (54.04)	343 (85.54)	58 (14.46)	
Sick leave	Yes	466 (64.1)	423 (90.77)	43 (9.23)	< 0.001
	No	261 (35.9)	211 (80.84)	50 (19.16)	
Duration of last stay in India	< 12 months	419 (56.47)	98 (82.35)	21 (17.65)	0.28
	12–23 months	241 (32.48)	531 (87.19)	78 (12.81)	
	24–35 months	50 (6.78)	44 (88.0)	6 (12.0)	
	36 months and later	32 (4.31)	25 (78.13)	6 (21.87)	
Reason for return	Holiday	348 (52.02)	330 (94.83)	18 (5.17)	< 0.001
	Personal problems^¶¶^	305 (45.59)	248 (81.31)	57 (18.69)	
	Others (end of contract, political issues)	16 (2.39)	11 (68.75)	5 (31.25)	
Going back^¶¶¶^	Yes	607 (81.59)	540 (83.59)	67 (11.04)	< 0.001
	No	137 (18.41)	106 (68.37)	31 (31.63)	
Current smoker	Yes	415 (55.41)	359 (86.51)	56 (13.49)	0.89
	No	334 (44.59)	290 (86.83)	44 (13.17)	
Current alcohol user	Yes	429 (57.12)	387 (90.21)	42 (9.79)	0.001
	No	322 (42.88)	263 (81.68)	59 (18.32)	
Smoke marijuana	Yes	32 (4.27)	25 (78.13)	7 (21.88)	0.15
	No	718 (95.73)	624 (86.91)	94 (13.09)	
Sex in last 6 months	Yes	502 (67.20)	452 (90.04)	50 (9.96)	< 0.001
	No	245 (32.80)	194 (79.18)	51 (20.82)	
Existing health problem^§^	Yes	114 (15.36)	76 (66.67)	38 (33.33)	< 0.001
	No	628 (84.64)	566 (90.13)	62 (9.87)	
Accessing healthcare	Difficult to access	197 (28.84)	153 (77.66)	44 (22.34)	< 0.001
	Easy to access	486 (71.16)	446 (91.77)	40 (8.23)	
Health expense coverage	Completely by employer	54 (11.04)	46 (85.19)	8 (14.81)	0.02
	Partially by employer	60 (12.27)	57 (95.0%)	3 (5.0%)	
	Completely by myself	375 (76.69)	302 (80.53)	73 (19.47%)	
Healthcare expense	Easy to manage	313 (42.07)	288 (92.01)	25 (7.99)	< 0.001
	Somehow manageable	311 (41.8)	269 (86.5)	42 (13.5)	
	Very difficult to manage	120 (16.13)	87 (72.5)	33 (27.5)	

Note: ^¶^, Given the column wise total proportion to compare the frequency distribution within each group; ^¶¶^, personal problem includes poor health and family issues; ^¶¶¶^, the question asked was ‘Do you intention to return to India for job?”; ^§^, the question asked was ‘Do you have any physical health problem? are you taking any medication currently?’

More than half of the participants were current smokers or alcohol drinkers [Table 2]. Overall, 15.4% reported that they currently had at least one disease or medical condition, and over three-quarters (76.7%) bore the treatment cost themselves [Table 2].

### Factors associated with psychological morbidity

The prevalence of psychological morbidity was 13.5% (CI: 11.2–16.1%). There were statistically significant differences in psychological morbidity by age, marital status, gender, education levels, ethnicity/caste, and income [Tables 1 and 2]. The data show that self-reported psychological morbidity was significantly higher in participants who were widowed/divorced (uPR = 3.48; CI = 1.46–8.29), older (uPR = 2.54, CI = 1.44–4.49), female = uPR 2.5; CI = 1.21–5.14), Dalit, Janajatis and others from the Terai (uPR = 3.6, CI = 2.26–5.7), religious minorities (uPR = 4.24, CI = 1.86–9.65), factory workers (uPR = 2.0, CI = 1.14–3.54), security guards (uPR = 1.89, CI = 1.06–3.36) and poor (monthly household income < 10,000 NR) [Table 3]. The adjusted regression analysis showed that participants aged 45 years or above were 2.74 times (aPR = 2.74, CI: 1.01–7.41) more likely to suffer from psychological morbidity than participants aged 25 years or younger [Table 3]. Similarly, the prevalence of psychological morbidity among Dalits and Janajatis from the Terai (aPR = 3.29, CI: 1.6–6.74) and minority groups (aPR = 3.64, CI: 1.02–13.14) was more than three times higher than the prevalence among the caste group Brahmin/Chhetri/Thakuri.

**Table 3:** Multivariable analysis for psychological morbidity

Characteristics		Prevalence ratio	
		uPR (CI)	aPR (CI)
Age	< 25 years	Reference	
	25–34 years	0.67 (0.38–1.18)	0.71 (0.29–1.72)
	35–44 years	1.06 (0.58–1.87)	0.82 (0.31–2.19)
	45 years and above	2.54 (1.44–4.49)**	2.74 (1.01–7.41)*
Gender	Male	Reference	
	Female	2.5 (1.21–5.14)***	0.77 (0.19–3.12)
Marital status	Unmarried	Reference	
	Married	0.79 (0.49–1.31)	0.69 (0.28–1.66)
	Others (widow, divorced)	3.48 (1.46–8.29)**	1.16 (0.14–9.9)
Education	No Schooling	Reference	
	Primary	0.58 (0.35–0.95)*	1.62 (0.72–3.65)
	Lower Secondary	0.33 (0.18–0.58)***	0.72 (0.26–1.98)
	Secondary	0.41 (0.22–0.77)**	1.2 (0.41–3.55)
	SLC and Higher	0.25 ((0.08–0.85)*	1.0 (0.18–5.76)
Ethnicity	Brahmin/Chhetri/Thakuri	Reference	
	Dalit and Janajatis Hill	0.77 (0.42–1.42)	0.45 (0.19–1.06)
	Dalit, Janajatis and others Terai	3.6 (2.26–5.7)***	3.29 (1.6–6.74)**
	Religious minorities	4.24 (1.86–9.65)**	3.64 (1.02–13.14)*
Occupation	Hotel worker	Reference	
	Factory worker	2.0 (1.14–3.54) *	1.12 (0.46–3.03)
	Watchmen	1.89 (1.06–3.36)*	1.35 (0.54–3.42)
	Others¶	1.31 (0.65–2.64)	1.65 (0.62–4.42)
¶¶Salary/month	< 10,000 INR	Reference	
	10,000–15,000 IR	0.69 (0.44–1.07)	0.88 (0.46–1.66)
	15,001 IR and more	0.37 (0.14–0.95)*	0.41 (0.14–1.19)
Sick leave	Yes	Reference	
	No	2.08 (1–38-3.12)***	2.4 (1.32–4.34)**
Reason for return	Holiday	Reference	
	Personal problems¶¶¶	3.61 (2.13–6.14)***	1.96 (1.01–3.83)*
	Others (end of contract, political issues)	6.04 (2.24–16.27)***	4.06 (1.08–15.28)*
Going back^§^	Yes	Reference	
	No	2.05 (1–34-3.14)*	1.23 (0.67–2.25)
Existing health problem^§§^	Yes	Reference	
	No	0.3 (0.2–0.44)***	0.50 (0.28–0.93)*
Current alcohol user	Yes	Reference	
	No	1.87 (1.26–2.78)***	0.99 (0.53–1.84)
Sex in last 6 months	Yes	Reference	
	No	2.08 (1.41–3.08)***	1.21 (0.62–2.37)
Accessing healthcare	Difficult to access	Reference	
	Easy to access	0.37 (0.24–0.57)***	0.53 (0.3–0.93)*

Note: *, < 0.05; **, < 0.01; ***, < 0.001

^¶^, other occupations were house servant, driver, agriculture workers; ^¶¶^, 1 Indian Rupee = 0.014 USD; ^¶¶¶^, personal problem includes poor health and family issues; ^§^, the question asked was ‘Do you have intention to return to India for job?”; ^§§^, the question asked was ‘Do you have any physical health problem? are you taking any medication currently?’

Self-reported psychological morbidity was not associated with the number of hours worked per day, the provision of days off, and the length of time stayed in India. However, there was a statistically significant association between psychological morbidity and having sick leave: not having sick leave doubled the rates of self-reported psychological morbidity (aPR = 2.4, CI = 1.32–4.34). Returning home for personal work (aPR = 1.96, CI = 1.01–3.83) and other reasons, for example, the end of one’s contract (aPR = 4.06, CI = 1.08–15.28), was also associated with high levels of psychological morbidity.

Similarly, participants with pre-existing health problems were twice (aPR = 2) as likely as other participants to report psychological morbidity. Current alcohol use (uPR = 0.53, CI = 0.35–0.79) and having had sex in previous six months (uPR = 0.48, CI = 0.32–0.71) were associated with low psychological morbidity in univariate analysis, but this association did not remain intact in multivariable analysis [Table 3]. Likewise, participants who had experienced no difficulties in accessing health facilities in the host country were likely to have a lower level of psychological morbidity (aPR = 0.53, CI = 0.3–0.93) than those without such easy access. Neither smoking tobacco nor smoking marijuana was significantly associated with an increase in self-reported mental health issues.

## Discussion

This study assessed the prevalence of and factors associated with psychological morbidity in Nepali cross-border migrants to India. We found that the burden of self-reported psychological morbidity was significant among the study population and associated with age, gender, ethnicity, education and income. The prevalence of psychological morbidity was significantly higher in participants with an existing health condition, those who had difficulty accessing health care, and those whose jobs had no provision for sick leave. This study, however, failed to incorporate some key variables that could affect the mental health status in migrant workers, such as social support, cohesion and cultural conflict in the host country.

The present study found that self-reported psychological morbidity (GHQ-12 score ≥ 6) was present in 13.5% of the participants. This finding is comparable to that of a study conducted in a similar setting with similar study participants [7], in which 24.4% of Bangladeshi and 15.1% of Nepali returnee migrants showed some level of distress (undichotomized GHQ-12 score > 20). The proportion of the participants having an undichotomized GHQ-12 score > 20 in our study is 20.2%. There is a paucity of Nepali studies that measure psychological morbidity using GHQ-12 tool with the same cut-off point (GHQ-12 score ≥ 6) as ours, but one study carried out in rural Nepal found that 9.8% of postnatal mothers experienced psychological morbidity [27]. Other studies estimated different psychological parameters such as anxiety and depression using tools other than the GHQ-12 and reported a wide range of findings [28–31]. A recent mental health prevalence survey in Nepal, for instance, found that 12.9% of Nepali had at least one mental disorders [32], while a survey of health problems of Nepali female migrant workers in the Middle-East and Malaysia reported that 8.7% women had mental health problems [33]. Likewise, studies conducted outside of Nepal also demonstrated the high burden of anxiety and depression among labour migrants [34]. The prevalence of anxiety and depression pooled from nine studies conducted among labour migrants in the USA, France and Uganda were 21% (CI:14–29%) and 20% (CI: 14–26%) [34] respectively. A recent systematic review identified 37 studies related to migration and mental health that had been conducted in low-and middle-income countries, where four of those studies reported the prevalence of depression to be between 3 and 51% [14].

The prevalence of psychological morbidity varied significantly by age, gender, ethnicity, education, occupation and household income among our study participants. It is generally observed that the rate of psychological illness is raised in advancing age [35] and is higher in females [15, 36, 37]. De Maio and Kemp also noticed an increased likelihood of deterioration of mental health among Canadian female immigrants [37]. The findings are consistent with a community-based study of a general population in Nepal [29], which found that age, gender and ethnicity were associated with higher depression and anxiety parameters [29]. One possible explanation for the high rate of psychological morbidity among Dalit and Janajati migrants from the Terai is the persistent poverty in the region. The Terai has a low Human Development Index (HDI) compared to the rest of the country (the mountain and hill regions) due to low literacy, income and life expectancy [38]. Furthermore, Nepali Dalits are more likely to be exposed to health, family, financial and political stressful life events than other castes/ethnic groups (Brahman, Chhetri and Janajatis) leaving them susceptible to depression and anxiety [29]. A wealth of literature demonstrated an inverse relationship between low socio-economic status and psychological wellbeing [15, 37, 39]. Setia et al. found that male immigrants in Canada with low incomes had nearly a two-fold risk (OR: 1.99, 95% CI: 1.38–2.86) of having severe psychological distress than immigrants in higher income categories [39].

This study did not find a statistically significant association between work-related conditions such as working hours and day off and psychological morbidity. However, the prevalence of psychological morbidity differed noticeably depending on whether or not migrants had a provision for sick leave in their last job. Similarly, rates of psychological morbidity did not vary much with the duration of migrants’ stay in India. That said, rates were significantly higher among those who returned home because of personal issues at home or the termination of contract in India. A study conducted among returnee Nepali and Bangladeshi migrants reported that study participants returned home mostly due to personal issues (poor health, family problems) and exhibited high psychological distress [7].

The findings indicated that pre-existing physical illness was linked to psychological morbidity in the study population. Moreover, most of the existing health problems among the study participants were related to chronic conditions such as diabetes, hypertension and chronic obstructive pulmonary diseases; all are diseases associated with psychological co-morbidity [40]. This finding is consistent with a study conducted among Romanian immigrants in Italy that showed the odds of experiencing psychological distress was 6 times higher among migrants suffering from chronic diseases than among those who were not [41].

In contradiction to the factors amplifying psychological morbidity, cultural closeness between Nepal and India could possibly help minimize cultural conflicts and might have a protective effect on the mental health of Nepali migrants [42, 43]. However, it is difficult to ascertain why living with family members (a proxy variable for family support) was not significantly associated with a lower rate of psychological morbidity.

Overall, the present study demonstrated that psychological morbidity is prevalent among Nepali cross-border migrants to India and particularly affects the special groups [44], such as older adults, women, Dalit and Janajati from marginalized areas and religious minority. Similarly, work and health related factors, for example, provision of sick leave, pre-existing physical illness and difficulty in accessing health services were significantly associated with psychological morbidity among the study participants. The findings suggest a need for resilience enhancing responses such as establishing legal frameworks to protect the rights of migrants including the right to health care and other social services in the destination country [1, 45, 46]. Evidence shows that culturally appropriate and contextualized psychosocial support interventions can also be effective for promoting mental health in a particular group of migrants [47]. Similarly, as suggested by Davies et al. [48], returnee migrants should also be re-integrated into the existing healthcare system for psychological support and proper management of other health conditions including tuberculosis. If they do not receive such care, the stigma attached to the explanatory model of mental illness prevalent in society may further delay in psychological help seeking among migrants [49]. The preliminary draft of Nepal’s migration health policy also speaks about monitoring migrants' health at different stages of migration and guides action to control the elevated risk of the cross-border transmission of communicable diseases. It is not clear how the policy helps address the health challenges incurred due to the current recordless, undocumented cross-border migration between Nepal and India or facilitates the integration and re-integration of these migrants into migrant specific and sensitive programs and policies in the destination and home countries.

This study has some methodological limitations. Despite the random selection of clusters, the survey applied non-probability sampling for selecting households. For this reason, the findings should be generalized beyond the study participants only with caution. Similarly, the GHQ-12 used in the study is a screening tool, not a diagnostic aid for psychological morbidity. Moreover, the tool was validated quite a long ago (in 1999) [23] and the high cut off value (≥6 GHQ-12 score) might have resulted in the underestimation of psychological morbidity in the study population. Likewise, information obtained from returnee migrants may not well represent current migrants working in India. As participants were selected from among those who declared themselves cross-border migrants, the sample was not very likely to have included the participants who had been involved in socially stigmatized and undesirable employment such as sex work. Lastly, because of the study’s cross-sectional design, establishing the causal relationship between the study and outcome variables is beyond its scope.

## Conclusion

This study assessed the prevalence and associated factors of psychological morbidity among Nepali cross border migrants who had returned from India. The findings showed the burden of psychological morbidity was significantly high among vulnerable groups such as women, the elderly, marginalized groups and minorities. The respondent’s self-reported psychological condition was associated with work and health related factors. In addition to offering insights into migrant’s psychological ill health, the findings point to the need for tailoring migrant specific mental health promotive interventions and strengthening the legal framework for providing rights and social securities to Nepali cross border migrants in India.

## Data Availability

The data used to support the findings of this study are available from the corresponding author upon request.
